# Energy Drink Consumption Among Physically Active Polish Adolescents: Gender and Age-Specific Public Health Issue

**DOI:** 10.3389/ijph.2024.1606906

**Published:** 2024-11-07

**Authors:** Dominika Granda, Olga Surała, Jadwiga Malczewska-Lenczowska, Beata Szczepańska, Anna Pastuszak, Radosław Sarnecki

**Affiliations:** ^1^ Department of Nutrition Physiology, Institute of Sport-National Research Institute, Warsaw, Poland; ^2^ Department of Biomechanics, Institute of Sport-National Research Institute, Warsaw, Poland; ^3^ Project Department, Institute of Sport-National Research Institute, Warsaw, Poland

**Keywords:** energy drinks, consumption, adolescents, physical activity, public health, nutrition, motives for health behaviours

## Abstract

**Objectives:**

To analyse the consumption of energy drinks (EDs) and the motives for their consumption among physically active adolescents in Poland.

**Methods:**

A nationwide survey study was conducted in 1,530 adolescents (10–14 years old) participating in extracurricular, organised sports activities. A computer-assisted web interview method was applied.

**Results:**

Nearly half (46.4%) of the respondents were ED consumers (significantly more boys than girls: 53.0% vs. 47.0%, p = 0.010). The percentage of ED consumers increased with age, from 27.2% in 10-year-olds to 65.4% in the group of 14-year-olds (p < 0.001). The motives and circumstances of ED consumption varied depending on gender: girls were more likely than boys to drink EDs to improve concentration during studying (17.1% vs. 8.8%, p < 0.001) and to stay awake (22.8% vs. 13.8%, p = 0.002), whereas boys more often than girls reported drinking EDs during physical activity (10.9% vs. 6.0%, p = 0.020).

**Conclusion:**

It is necessary to implement gender-diversified educational campaigns on negative health effects of EDs, targeting children, parents and teachers, as consumption of EDs has been identified as a significant public health problem in Poland.

## Introduction

Energy drinks (EDs) are non-alcoholic functional beverages with a stimulating effect, intended for adults subjected to increased mental and/or physical stress [1]. These drinks are often advertised as reducing fatigue, improving concentration, and providing energy. EDs should not be confused with isotonic drinks, which are designed to replenish water and electrolytes lost in sweat to maintain proper hydration, as well as to provide small amounts of carbohydrates to refuel during and after exercise [2]. In contrast, a characteristic feature of EDs is their high caffeine content (>150 mg/L), which is not present in isotonic drinks. Other substances which can be found in EDs include taurine, glucuronolactone, B vitamins, L-carnitine, sucrose, minerals and herbal supplements such as ginseng, guarana, yerba mate, cocoa, kola nut, and ginkgo biloba [3]. The effects of caffeine, a crucial ingredient of EDs, on the human body are mediated by several mechanisms, including: antagonism of adenosine receptors, inhibition of phosphodiesterase, intracellular mobilization of calcium ions, and antagonism of benzodiazepine receptors. Due to the caffeine content, EDs may provide beneficial effects such as improved psychomotor performance (reaction time, memory, concentration) [4, 5]. Simultaneously, caffeine increases heart rate, dilates blood vessels, and increases thermogenesis and lipolysis [6].

Caffeine is considered well studied in terms of exercise performance. In studies on both professional athletes and amateurs, its effectiveness has been demonstrated by enhanced aerobic endurance and its ergogenic effects on cognitive function [7]. As stated in the recently published position stand by the International Society of Sports Nutrition (ISSN), EDs can also enhance acute aerobic exercise performance [8]. Nevertheless, according to the above-mentioned position stand, EDs are not recommended for children (aged 2–12 years old) in relation to physical exercise. Adolescents who consider drinking EDs around training should exercise caution and ask for parental guidance due to insufficient data on caffeine safety in this population. While the effects of caffeine have been extensively studied in adults, with both positive and negative outcomes observed depending on dosage, genotype, and other factors, conducting similar experimental studies in children poses ethical challenges. According to our knowledge, so far, only one such study has been performed [9]. Given that EDs are relatively recent products on the market, the long-term consequences of their regular consumption remain poorly understood. Observational studies conducted among children and adolescents have limitations that prevent the confirmation of definite cause-and-effect relationships. Therefore, due to the lack of sufficient data on the relationship between caffeine intake and health outcomes in the paediatric population, experts from the European Food Safety Authority (EFSA) have yet to determine a safe daily dose of caffeine for children [10].

Childhood and adolescence are critical periods for brain development, particularly in processes such as myelination of white matter and changes in grey matter volume, which reaches its adult size between ages 20 and 25. EDs are suspected of potentially causing damage in these areas of the brain [11]. Consumption of EDs among children and adolescents may increase the risk of cardiovascular, neurological, psychiatric, gastrointestinal, metabolic, and other disorders [12–14]. Adolescents who regularly consume EDs have reported higher frequency of stress, nervousness, anxiety, and depression symptoms [15, 16]. In teenagers, aside from the potentially direct impact of stimulants on brain function, it has been demonstrated that these substances affect sleep duration and quality. Adolescents who frequently consumed caffeine-containing beverages were more likely to not meet the recommended sleep duration guidelines [17–20], including experiencing insomnia [21, 22]. However, it is not only the high caffeine content in EDs that may be associated with negative health outcomes. It should also be emphasized that EDs contain a high amount of sugar, which can negatively impact oral health—a concern that is already prevalent among children and adolescents in Poland [23]. Regular consumption of such beverages may also contribute to an excessive energy intake, leading to the development of overweight and obesity, along with all the associated health consequences [24]. This is supported by research findings indicating that ED consumption among adolescents was associated with a higher body mass index [25].

Considering the above reports, it may be surprising that the marketing of EDs is aimed not only at people involved in sports and esports, but also at children and adolescents using advertising strategies that appeal to their interests, lifestyles, and social circles [26]. By sponsoring popular events such as skateboarding, snowboarding, and gaming tournaments, ED companies align their brands with activities that resonate with younger demographics. These events often attract young people who are passionate about action sports or gaming culture, creating a direct connection between the brand and their interests. Many countries have already implemented legal measures aimed at limiting the consumption of EDs among children and adolescents. In Sweden, Denmark, Argentina, and as of January 2024, also in Poland, there are restrictions on the sale of EDs to underaged persons, similar to those applied to alcohol sales. Other actions include additional taxation affecting product pricing, bans on sales in schools, and specific guidelines regarding packaging and labelling [27].

In order to understand why adolescents consume EDs, it is crucial to identify the driving factors. Despite national discussions, there is a lack of systematic, up-to-date data regarding this problem among Polish youth, with details on consumption patterns evolving over time. This study aimed to analyse the consumption of EDs and the motives for their consumption among adolescents involved in sports in Poland.

## Methods

### Study Design

We conducted a nationwide survey with the web-based survey method between October and November 2022 (prior to the ban on the sale of EDs to underaged persons). The survey was developed for primary school students in grades 1–8 aged between 7 and 14, participating in extracurricular, organised sports activities within the School Sports Club (Polish *Szkolny Klub Sportowy*, SKS) programme. SKS is a Polish systemic activity aimed at primary and secondary school students. Although the purpose of the survey was primarily to assess the attitudes and opinions about physical activity and participation in the SKS programme, it also included questions regarding the consumption of EDs, as this has been identified as a significant public health problem among children and adolescents in Poland [28–31]. Since the physical activity survey was sent to all children participating in SKS (7–14 years), questions about ED were also sent to all children. However, later it was decided to analyse only data from children aged 10–14 because the survey may not have been sufficiently adapted to the abilities of younger children. Moreover, the size of this group was the smallest, thus increasing the risk of selection bias. The electronic version of the survey was distributed to randomly selected primary schools participating in the SKS programme. The selection of the sample was purposeful as the survey was addressed to children and adolescents participating in additional physical education classes. The link to the online survey was sent to the individual accounts of teachers coordinating the SKS programme in randomly selected schools in Poland. In total, the SKS programme covers 8,255 schools throughout Poland. Surveys were sent to teachers from 825 randomly selected schools, taking into account the population in individual voivode ships. Assuming that one teacher leads a group of an average of 12 children, the survey was made available (via the teacher) to 9,900 students. Teachers were asked to allow the survey to be completed individually during additional SKS sports classes. Students completed the survey anonymously, but the teacher was present in the room and could help in case of doubts. We received completed questionnaires including questions regarding the consumption of EDs from 1741 students. Our study was reviewed and approved by the Ethics Committee of the Institute of Sport—National Research Institute, Poland (KEBN-23–84-TMK). This study was carried out in accordance with the Declaration of Helsinki (2000) of the World Medical Association.

### Data Collection Questionnaire and Data Analysis

The survey questions were partly based on questions from a validated questionnaire published by EFSA in 2013 [10] – a shortened version was created in which we included questions about the frequency, motives and circumstances in which EDs were consumed. Their short definition preceded the unit with questions about EDs. The questions were closed-ended, both single and multiple-choice. We asked, among other questions, whether, in the respondents’ opinion, isotonic and EDs are the same products, and on this basis, we determined whether this population correctly recognized EDs. The question: “Have you consumed energy drinks in the last 3 days?” was asked to divide the respondents into ED consumers and non-consumers. The respondents who answered “I never consume energy drinks” were classified as non-consumers and could finish completing the survey at this point. Those who answered “yes” or “no” were classified as ED consumers. In further questions, we asked the ED consumers about the frequency of ED consumption in the previous year (single-choice question). The following options were available: “less than once a month,” “1–3 times a month,” “1-2 times a week,” “3-4 times a week,” “5-6 times a week,” “once a day,” “twice a day,” “3 or more times a day.” Frequency categories were adapted from the Food Frequency Questionnaire developed at Karolinska Institute [32]. The ED consumers were divided into three categories based on the frequency of consumption: infrequent consumers (those who chose the answers “less than once a month” and “1–3 times a month”), frequent consumers (those who chose the answers “1-2 times a week” and “3-4 times a week”), and highly frequent consumers (those who chose the answers “5-6 times a week,” “once a day,” “twice a day,” “3 or more times a day”). We also asked about the most frequently chosen product brands and packaging size; which allowed us to calculate average daily caffeine intake (expressed as milligrams per day) from EDs. Continuous variables were presented as the means (x̄) and standard deviations (SD), whereas categorical variables were reported as counts and percentages. Means of continuous variables were compared using Student’s t-test for independent samples, whereas Pearson’s chi-square test (χ^2^) was used to examine categorical study variables. To identify the significant pairs between groups, multiple column comparisons using RxC analysis with Benjamini-Hochberg correction were applied. For selected dependent variables, the adjusted odds ratio (aOR) was calculated using the multi-nominal logistic regression model. To determine predictors of being an ED consumer, age-adjusted odds ratios (ORs) with 95% confidence intervals (95% CI) were calculated using logistic regression models. A p-value <0.05 was considered statistically significant. Statistical analysis was performed using Statistica (Version13) software.

## Results

### Study Group Characteristics

In total, 1741 students responded to the questions included in the survey regarding the frequency of ED consumption. Due to the small number of the youngest children (n = 211), we decided to exclude them from the study and focus on those 10 years old and older. The final study sample consisted of 1,530 adolescents aged 10–14 years, with a mean age of 12.0 ± 1.2 years. Background characteristics are presented in Table 1. There was an approximately even distribution between boys and girls, and nearly 2/3 of the participants lived in rural areas (65.0%). As mentioned previously, all students included in the study were attendees of sports classes. Almost 40% of responders had participated in the SKS programme for more than a year. Of all participants, 46.4% were recognized as ED consumers. Slightly more than a half (57.8%) of adolescents were aware that EDs and isotonic drinks are different products, while 36.7% of participants found it difficult to differentiate them. The inability to distinguish between these beverages was found in 5.4% of the respondents.

**TABLE 1:** Descriptive characteristics for total sample (n = 1,530) (Poland, 2022).

Variable	N	%
Age group		
10	206	13.5
11	353	23.1
12	458	29.9
13	308	20.1
14	205	13.4
Gender		
Female	774	50.6
Male	756	49.4
Residency		
Urban	536	35.0
Rural	994	65.0
Sports classes attendance		
Started this year	441	28.8
Since last year	482	31.5
Longer	607	39.7
ED recognition^a^		
Yes	884	57.8
No	83	5.4
Difficult to say	562	36.7
ED consumer		
Yes	710	46.4
No^b^	820	53.6

EDs, energy drinks.

^a^ Response to question “Is an energy drink and an isotonic drink the same kind of drink?”.

^b^ Reported never consuming energy drinks.

### Characteristics of ED Consumers and Differences Between Consumers and Non-Consumers

**FIGURE 1:**
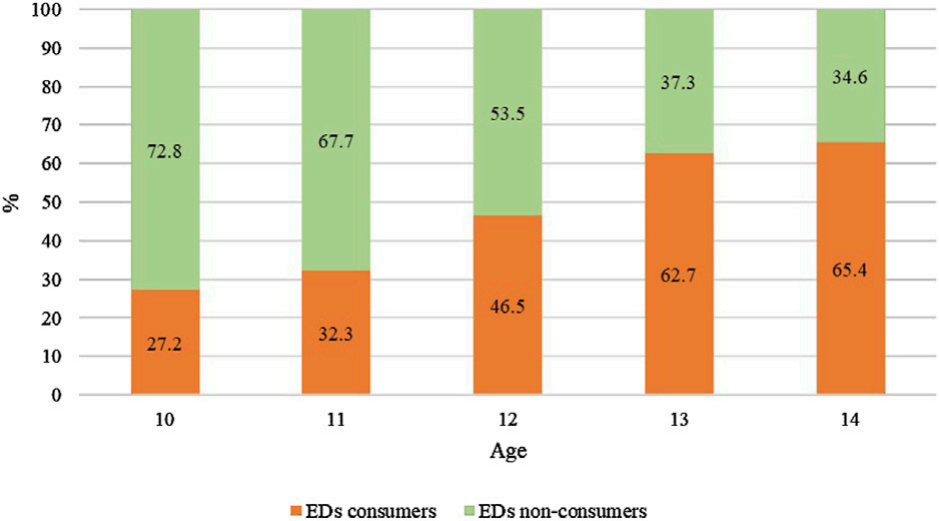
Distribution of energy drink consumers among various age groups (Poland, 2022).

The ED consumer group was significantly dominated by boys (53.0% vs. 47.0% girls, respectively) and those aged 12 and 13 (Table 2). The age-adjusted model of logistic regression also showed that male gender increased the likelihood of drinking EDs by 35% (Table 3). The percentage of EDs consumers increased with age, from 27.2% in the group of 10 years old to 65.4% in the group of 14 year olds (Figure 1). It was also confirmed by the logistic regression analysis that the probability of being a consumer of EDs increased significantly with age (Table 3). Recognition of EDs was significantly higher in the ED consumer group compared to non-consumers (65.6% vs. 51.0% respectively). In comparison to respondents who were able to differentiate EDs from isotonic drinks, those who believed they were the same drinks had significantly higher odds of being an ED consumer (OR: 2.28, 95% CI: 1.38–3.77). Place of residency (rural/urban) was not associated with drinking EDs. The detailed characteristics of ED consumers (n = 710) are summarized in Table 4. The most often chosen frequency of consumption of EDs was “less than once a month” (56.9%), followed by “1–3 times a month” (20.8%). The percentage of adolescents with daily consumption of EDs was relatively low (3.6%). Among EDs consumers, the largest group were infrequent (77.7%), followed by frequent (17.3%) consumers. Highly frequent consumers were a minority (5%). The main reasons for using EDs were their taste (52.8%), then “need for energy” (35.1%), aiming to stay awake (18.0%) and improving concentration during studying (12.7%). EDs were mainly consumed with friends and/or during parties (45.8%), as well as at home (29.6%). A lower percentage of ED consumers reported drinking them at school (9.0%) and during training or recreational physical activity (8.3%).

**TABLE 2:** Descriptive characteristics of energy drink consumers and non-consumers (Poland, 2022).

	ED consumers (n = 710)	ED non-consumers (n = 820)	p-value (χ^2^)
Age – years (x̄ ± SD)	12.3 ± 1.2	11.7 ± 1.2	<0.001^a^
Gender n (%)			
Female	334 (47.0)	440 (53.7)	0.010
Male	376 (53.0)	380 (46.3)	
Age n (%)			
10	56 (7.9)	150 (18.3)	<0.001
11	114 (16.1)	239 (29.1)	
12	213 (30.0)	245 (29.9)	
13	193 (27.2)	115 (14.0)	
14	134 (18.9)	71 (8.7)	
Residency n (%)			
Urban	240 (33.8)	296 (36.1)	0.348
Rural	470 (66.2)	524 (63.9)	
Energy drink recognition^b^ n (%)			
Yes	466 (65.6)	418 (51.0)	<0.001
No	58 (8.2)	25 (3.0)	
Difficult to say	185 (26.1)	377 (46.0)	

EDs, energy drinks.

^a^ Student’s t test.

^b^ Response to question “Is an energy drink and an isotonic drink the same kind of drink?”.

**TABLE 3:** Logistic regression of energy drink consumption by selected socio-demographic determinants (n = 1,530) (Poland, 2022).

Study factor	OR (95% CI)
	EDs consumers vs. non-consumers
Age (years)	1.61 (1.47–1.75)*
Gender	
Girls	1.00
Boys	1.35 (1.09–1.66)*
Residency	
Rural	1.00
Urban	0.82 (0.66–1.02)
EDs recognition	
Yes	1.00
No	2.28 (1.38–3.77)*
Difficult to say	0.50 (0.40–0.63)*

Age-adjusted model, CI, confidence interval; OR, odds ratio, *p-values<0.05.

**TABLE 4:** Characteristics of energy drink consumers (n = 710) (Poland, 2022).

	N	%
Frequency of ED consumption among consumers		
<once a month	404	56.9
1–3 times/month	148	20.8
1–2 times/week	95	13.4
3–4 times/week	28	3.9
5–6 times/week	10	1.4
Once a day	9	1.3
Twice a day	9	1.3
3 or more time a day	7	1.0
ED consumer types in terms of frequency of consumption		
Infrequent*	552	77.7
Frequent**	123	17.3
Highly frequent***	35	5.0
Reasons for using EDs^a^		
I like the taste	375	52.8
I need energy	249	35.1
To stay awake	128	18.0
To improve concentration while studying/learning	90	12.7
To improve physical performance in training	52	7.3
To boost metabolism	21	3.0
Circumstances of ED consumption^a^		
with friends, during parties	325	45.8
at home	210	29.6
at school	64	9.0
during training, recreational physical activity	59	8.3
at restaurants, bars	20	2.8
in clubs	12	1.7
with alcohol	8	1.1

EDs, energy drinks, *reported frequency of consumption from <once a month to 1–3 times a month, ** reported frequency of consumption from 1 to 2 times a week and 3-4 times a week, *** reported frequency of consumption from 5 to 6 times/week to 3 or more time a day.

^a^ multiple choice question.

### Daily Caffeine Intake From EDs Among Consumers

Data on caffeine intake from EDs is provided in the Supplementary File. The average daily caffeine intake from EDs in the consumer group was 20.8 ± 81.2 mg (Supplementary Table S1). The mean daily intake was significantly higher among boys compared to girls (p < 0.05). The highest caffeine intake was observed in the 13-year-old group, while the lowest was found in the 10-year-old group (41.7 ± 142.1 mg and 6.3 ± 11.9 mg, respectively). No statistically significant differences were identified between consumers from urban and rural areas. Additionally, no differences in caffeine consumption from EDs were observed across varying levels of knowledge about EDs.

### Motives and Circumstances of EDs Consumption by Different Age Groups

The results presented in Table 5 indicate that in all age groups of EDs consumers, except for 14-year-olds, good taste was the most common reason for reaching for these beverages (ranging from 57.5% in 13-year-olds to 48.2% in 11-year-olds), followed by the need for energy (ranging from 17.9% in 10-year-olds to 35.8% in 13-year-olds). In the case of 14-year-olds, the contribution of both reasons was similar: 47.0% for the need for energy and 45.5% for the good taste. The answers “I need energy” and “To stay awake” were chosen significantly more often in older age groups compared to the group of 10-year-olds (p-value = 0.002 and p-value = 0.021 respectively), while there were no statistically significant differences between age groups for other motives. It was also observed that wanting to improve concentration while studying was more prevalent among 12-year-olds and older children than in 10 and 11-year-olds; however, the difference was not statistically significant. Improving physical performance in training was a motive for 1.8% of 10-year-olds to 9.6% of 11-year-olds.

**TABLE 5:** Motives and circumstances of energy drink consumption among consumers–age and gender perspective (n = 710) (Poland. 2022).

	Age							Gender		
	Total	10 years (n = 56)	11 years (n = 114)	12 years (n = 213)	13 years (n = 193)	14 years (n = 134)	p-value (χ^2^)	Boys (n = 376)	Girls (n = 334)	p-value (χ^2^)
Reasons for using EDs (%)										
I like the taste	52.8	55.4	48.2	54.9	57.5	45.5	0.195	51.1	54.8	0.321
I need energy	35.1	17.9a	31.6ab	33.3b	35.8bc	47.0c	0.002	35.9	34.1	0.621
To stay awake	18.0	3.6a	16.7b	16.9b	20.7b	23.1b	0.021	13.8	22.8	0.002
To improve concentration while studying	12.7	8.9	5.3	13.6	15.5	14.9	0.072	8.8	17.1	<0.001
To improve physical performance in training	7.3	1.8	9.6	8.5	7.3	6.0	0.380	8.2	6.3	0.312
To boost metabolism	3.0	0.0	4.4	3.8	3.1	1.5	0.405	3.7	2.1	0.201
Other	21.7	26.8	24.6	23.9	17.6	19.4		22.6	20.7	0.530
Circumstances of ED consumption (%)										
with friends, during parties	46.3	30.4a	32.5ab	46.9c	58.0d	47.0cd	<0.001	44.4	48.5	0.276
at home	30.1	35.7	33.3	27.2	33.7	24.6	0.184	30.9	29.3	0.662
at school	9.4	5.4a	5.3a	4.2a	8.8a	23.9b	<0.001	7.5	11.7	0.054
during training, recreational physical activity	8.6	5.4	5.3	7.5	10.4	11.9	0.286	10.9	6.0	0.020
at restaurants. bars	2.8	1.8	0.0	2.8	4.7	3.0	0.204	4.0	1.5	0.045
in clubs/disco	1.8	5.4	0.0	0.9	3.1	1.5	0.182	2.1	1.5	0.532
with alcohol	1.1	1.8	0.9	0.0	2.1	1.5	0.352	1.6	0.6	0.210
others	28.9	32.1	36.0	29.1	25.9	25.4	0.201	28.7	29.0	0.923

EDs, energy drinks, different letters indicate significant difference (p < 0.05).

Energy drinks were consumed on several different occasions, which varied significantly among age groups, as presented in Table 5. Adolescents aged 12–14 years old drank EDs mainly during meetings with friends and parties (from 46.9% in 12-year-olds to 58.0% in 13-year-olds) significantly more often (p < 0.001) than 11- and 10-year-old students (32.5% and 30.4%, respectively). The percentage of subjects consuming EDs at school was six times higher in the oldest age group (14-year-olds) than in 12-year-olds (23.9% vs. 4.2% respectively) and almost three times higher than in 13-year-olds (8.8%, p < 0.001). Other circumstances of ED consumption did not vary among age groups. It was however observed that the popularity of using EDs during training or recreational physical activity increased with age (from 5.4% in 10- and 11-year-olds to 11.9% in 14-year-olds). Single respondents from all age groups (except for 12-year-olds) reported combining EDs with alcohol.

### Motives and Circumstances of EDs Consumption by Gender

The main motives for ED consumption (good taste and need for energy) were similar among boys and girls who were classified as ED consumers (Table 5). Girls more often than boys reported using EDs to stay awake (22.8% vs. 13.8% respectively, p = 0.002). Girls were also more likely than boys to use EDs in order to improve concentration during studying (17.7% vs. 8.8% respectively, p < 0.001). There were no statistically significant differences between genders in terms of other motives. Consumers of EDs, regardless of gender, were also similar in terms of the most common circumstances of using these beverages (with friends and at home). Significantly more surveyed boys than girls reported drinking EDs during training or recreational physical activity (10.9% vs. 6.0% respectively, p = 0.020), and when going to a restaurant or a bar (4.0% vs. 1.5% respectively, p = 0.045). Slightly more girls than boys reported drinking EDs at school (11.7% vs. 7.5% respectively, p = 0.054), but this difference was only of borderline statistical significance. There were no statistically significant differences between genders regarding other occasions for using EDs such as going to a club or mixing EDs with alcohol, which could be because very few respondents chose this answer.

## Discussion

In the present study we found that nearly half (46.4%) of the participants aged 10–14 years were ED consumers. These results are different from those published in 2013 by EFSA experts, who found that 73% of teenagers in Poland consumed EDs [10]. This discrepancy may be due to the fact that Zucconi et al. (2013) included adolescents aged 10–18 in their study group, while in this study subjects up to 14 years of age were examined. At the same time, the results of our study showed that the frequency of consuming EDs increased with age, which was consistent with the results of others [33]. It can also be assumed that the consumption of EDs has changed over the last 10 years, but the study by Zucconi et al. was the only one available which was conducted on a large sample (4,368) and used a validated tool. Results more similar to ours come from the 2021 National Study on the Diet and Nutritional Status of the Polish population, on a representative sample: EDs were consumed by 35.7% of boys and 27.4% of girls aged 10–17 [34]. However, it should be noted that this study assessed the overall diet, so the question about EDs was one of many, which could affect the accuracy of the data obtained, and the age range of the subjects was wider. It is also worth mentioning the results of the multi-centre project Health Behaviour in School-aged Children (HBSC), conducted, among other countries, in Poland [35]. In 2022, in a representative sample of 5,395 children aged 11, 13, and 15, the percentage of ED consumers was 46.7%, which closely aligns with our findings. According to other studies conducted in Poland in recent years, the percentage of adolescent ED consumers varied in different regions of the country: from 54% in Lublin province [29] to 89% in Subcarpathia and Lesser Poland provinces [28]. In our study, we aimed to present data on ED consumption by province; however, due to the small sample sizes within individual provinces, we decided to present the results for the entire group as a whole. Compared to global data, the percentage of subjects classified as ED consumers in this study seems relatively low: in the United States, 64.3% of teenagers aged 13–17 reported occasional consumption of energy drinks [36], while in Canada 62% did so (among students aged 12–18 years) [37]. Based on the results published by Nuss et al. [38], who studied ED consumption among Australian adolescents it can be concluded that more Polish than Australian students (aged 12–17 years, mean age 14.7 years) used EDs (46% vs. 24%). According to the previously mentioned EFSA report [10], which included data from a total of 52,000 inhabitants of the European Union, 68% of teenagers used EDs – ranging from 48% in Greece to 82% in the Czech Republic. While discussing the popularity of EDs among adolescents according to different authors, some discrepancies in the findings occur, probably due to methodological differences between studies in terms of defining an ED consumer. In the present study we decided to adopt a broad definition, as we included both individuals with daily consumption of ED and those who consumed them once a month or less often. A similar definition was also applied in the EFSA report [10]. Another potential explanation for the lower percentage of ED consumers in our study compared to others may be the age of studied subjects – in the present study we focused on 10–14-year-olds, while most other authors included older students up to 18 years of age. This is important because, as shown in our and other studies [39], ED consumption increased with age.

Male participants in our study were more likely to drink EDs than female participants (53.0% vs. 47.0%), which was in line with other studies conducted in Poland [28, 29], as well as in other countries such as Norway [40], Slovakia [33, 41] and Germany [42]. In the above-mentioned HBSC study regarding Polish youth, the frequency of EDs consumption among girls and boys reporting daily consumption of EDs was similar (7.8% and 6.5%, respectively) [35]. However, it should be emphasized that this study compared groups consuming EDs every day. Notwithstanding, future research should not omit girls, especially considering the results indicating that the increase in popularity of EDs in 2018 compared to 2014 was more pronounced in the group of girls than boys [39].

Consistent with previous studies from Poland [30], we did not find any differences in ED consumption among adolescents living in urban and rural areas. This contrasts with the study by Błaszczyk-Bębenek et al. [28], which detected higher ED consumption among children and adolescents living in bigger cities compared to those living in smaller cities.

The average daily caffeine intake from EDs among consumers in our study was 20.8 ± 81.2 mg, which was twice as high compared to a study from India, where 300 adolescents were surveyed [43]. The authors did not observe any differences in caffeine consumption between boys and girls; however, in our study, boys consumed significantly more caffeine from EDs than girls. In a study of the Polish population [44], it was found that the average daily caffeine intake from EDs was 14.31 ± 43.96 mg; it should be emphasized, however, that the majority of participants were adults. In contrast, in the study by Jia et al. [45], the average daily caffeine intake from all sources among consumers of caffeine-containing snacks aged 6–17 years was 21.6 mg. However, the main sources of caffeine were tea and coffee, while energy drinks and other carbonated beverages ranked third, providing 15% of the total caffeine intake. In the Korean population aged 15–18 years, caffeine intake from all sources was estimated at 30 mg per day, and the contribution of EDs to overall caffeine intake in the entire population was estimated at 0.8% [46]. It is important to emphasize that in our study, we assessed caffeine intake exclusively from EDs, and therefore the relatively high values obtained are particularly concerning.

Our results indicate that good taste was the most common motive for consumption among all age groups of ED consumers, except for 14-year-olds. Only 14-year-olds most often stated that the motive for drinking EDs was “the need for energy.” In general we observed a tendency for motives such as “need for energy” or “need to stay awake” to become more popular with age. This implies the need to include older age groups in future research as consumption motives seem to change, probably due to increasing expectations and requirements at school and from the peer environment. Other cross-sectional studies have also demonstrated this growing trend concerning the “need to stay awake” motive [10]. From the gender perspective, girls were more likely than boys to report using EDs to stay awake and to improve concentration during studying. The desire to stay awake may be connected with the fact that sleep disturbances seem more common among adolescent girls than boys [47]. Although we did not study the aspect of sleep quality, our results may suggest that the proper sleep pattern in girls could have been disturbed by drinking EDs: after using EDs during the day, problems with falling asleep might be more likely to occur, and caffeine may lower sleep quality [48]. After a night which did not bring the expected rest, drinking EDs on the following day is aimed at eliminating fatigue and maintaining concentration at school, and the cycle repeats. The message sent from educational campaigns and parents and teachers to children should clearly indicate that fatigue ought to be interpreted as a signal to rest.

Although other motives were chosen less frequently, attention should also be paid to children using EDs to improve physical performance during training (from 1.8% to 9.6% in various age groups in the present study). According to the previously cited position of the ISSN [8], EDs are not recommended for physically active adolescents under 12 years of age. ED use under parental supervision may be considered in those above this age. Our study did not assess the level of parental control, although it can be assumed that it varies greatly depending on the case, because not all parents are duly interested in the nutritional choices of their children [49]. The frequent consumption of EDs at home observed in our study may corroborate this.

As regards the circumstances of ED consumption, respondents, regardless of age, reported drinking them mainly with friends (46.3%) and at home (30.1%), less often at school (9.4%) and during training or recreational physical activity (8.6%). Even though these results are similar to those of other authors, it is difficult to compare them due to slightly different answer options. German adolescents (aged 9–19 years, mean age 13.1 years) used EDs mainly during parties (59%), and nearly 8% reported also drinking EDs while working out [42], which is in line with our results. A higher percentage (14.6%) of adolescents drinking EDs in the context of physical activity was found in the Spanish population [50]. It is worth mentioning that consuming meals and drinks, aside from their nutritional function, also serves a social function. For young people, the need to belong to a group is more important than for adults, so it can be assumed that the consumption of EDs by peers may encourage others to drink these beverages [51]. Another disturbing aspect is the low level of knowledge of young people about EDs: in our study, over 40% of total participants had problems distinguishing an isotonic drink from an ED. Other authors also have highlighted the problem of insufficient knowledge. For example, in the study by Kwiatkowska et al. [29], only 6% of respondents were aware that EDs may be harmful to young people. This is particularly alarming given that it has been shown in observational studies that excessive consumption may result in tachycardia, vomiting, cardiac arrhythmias, seizures, disrupted sleep patterns and even physiological dependence [52].

### Strengths and Limitations

There are some strengths and limitations of our study that should be taken into account. Although care was taken to ensure an even distribution of girls and boys and to include children living in urban and rural environments, our study group was quite specific: children attending additional sports activities. Hence, extrapolating the results to the entire population of young people in Poland is not fully justified*.* The choice of such a group may be questionable, but we aimed to assess the consumption of EDs in this group as physically active children and adolescents may consume isotonic drinks more often than their non-exercising peers. Studies conducted in other countries have shown that confusion between these two products is common among children and adolescents [2, 53], which was also confirmed in our study. Despite the research being conducted in individuals from various backgrounds, diverse in terms of urbanization, more respondents were inhabitants of rural areas (n = 994, 65%), an environment in which sports activities within SKS are usually the only available and free form of organized physical activity for children and adolescents. On the other hand, the place of residency (urban vs. rural) was not significantly related to ED consumption. The data on ED consumption, although collected anonymously, which increases the chances of honest answers, were self-reported, which makes them prone to self-reporting bias. We realize that in order to thoroughly analyse all factors that could potentially influence ED consumption, it would be necessary to collect more data, for example data on socio-economic status. The number of questions included in the survey was limited because it was part of another study and it was not our intention to significantly extend the time needed to complete the survey. Among the strengths of the current study is the relatively large study group, which covered the whole geographical area of Poland (all provinces).

### Conclusions

The present results indicate that consumption of EDs is relatively widespread among physically active Polish adolescents. Therefore we believe this issue should be considered an essential public health problem. Adolescents drink EDs primarily because of their taste, and a large percentage of them do not distinguish EDs from isotonic drinks. This indicates an urgent need to introduce preventive and wide-ranging educational activities, involving not only children, but also parents/legal guardians and teachers. As demonstrated in this study, the motives and circumstances of ED consumption varied depending on gender: girls were more likely than boys to drink EDs in the context of learning and sleeping, whereas boys more often than girls reported drinking EDs during physical activity. It suggests the need to diversify educational campaigns on EDs targeted at girls and boys. This conclusion is further supported by the fact that the issue of ED consumption concerned boys to a greater extent than girls. The present results confirm the validity of recently undertaken legal actions to limit the possibility for people under 18 years of age to purchase EDs in Poland. It should be underlined that this study was conducted prior to the implementation of the ban on the sale of EDs to minors. Therefore, it is necessary to repeat the study on a similar population of children and adolescents involved in sports in order to assess the effectiveness of this ban.
