# Supplementary material for: Energy Drink Consumption Among Physically Active Polish Adolescents: Gender and Age-Specific Public Health Issue
Source: Int J Public Health. 2024 Nov 7;69:1606906. doi: 10.3389/ijph.2024.1606906 (PMC11580739; doi:10.3389/ijph.2024.1606906)
Supplement: Supplementary file 1 [file Table1.docx]

Supplementary Materials

Journal: International Journal of Public Health

Title: “Energy Drink Consumption among Physically Active Polish Adolescents: Gender- and Age-Specific Public Health Issue”

Table S1 Average daily caffeine intake from energy drinks among consumers (n=710), data expressed as x̄ ± SD (Warsaw, Poland. 2022)

|  | Daily caffeine intake from EDs [mg] | p-value |
| --- | --- | --- |
| All consumers | 20.8 ± 81.2 | - |
| Gender |  |  |
| Female | 20.3 ± 72.1 | 0.019^#^ |
| Male | 21.3 ± 88.6 |  |
| Age |  |  |
| 10 | 6.3 ± 11.9 |  |
| 11 | 10.2 ± 25.0 |  |
| 12 | 8.9 ± 21.6 | 0.001* |
| 13 | 41.7 ± 142.1 |  |
| 14 | 24.9 ± 60.2 |  |
| Residency |  |  |
| Urban | 21.1 ± 86.9 |  |
| Rural | 20.7 ± 78.3 | 0.782^#^ |
| Energy drink recognition |  |  |
| Yes | 25.0 ± 69.4 | 0.851* |
| No | 19.2 ± 80.8 |  |
| Difficult to say | 22.0 ± 83.1 |  |

EDs – energy drinks, #U Mann-Whitney, *Kruskal-Wallis
